# Multidimensional immunotherapy for dry eye disease: current status and future directions

**DOI:** 10.3389/fopht.2024.1449283

**Published:** 2024-11-01

**Authors:** Duliurui Huang, Zhijie Li

**Affiliations:** ^1^ Department of Ophthalmology, People’s Hospital of Zhengzhou University, Henan Provincial People’s Hospital, Zhengzhou, China; ^2^ Henan Eye Institute, Henan Eye Hospital, Henan Provincial People’s Hospital, People’s Hospital of Henan University, People’s Hospital of Zhengzhou University, Zhengzhou, China

**Keywords:** Dry Eye Disease, immunotherapy, gene therapy, stem cell therapy, nanotechnology

## Abstract

Dry Eye Disease (DED) is a multifactorial condition driven by tear film hyperosmolarity, immune dysregulation, and neuro-immune interactions. The immune system plays a central role in its pathogenesis, influencing both inflammation and ocular surface damage. While traditional immunotherapies like anti-inflammatory agents and immunosuppressants offer symptom relief, their long-term use is limited by side effects. This review focuses on emerging immunotherapies, including biologics, stem cell therapy, gene therapy, nanotechnology, and exosome-based treatments, all of which hold promise in modulating immune responses and promoting tissue repair. The relationship between the ocular microbiome and DED is also explored, with an emphasis on personalized immunotherapy. Key challenges for future research include identifying novel therapeutic targets, optimizing clinical translation, and evaluating the long-term efficacy of these innovative treatments.

## Highlights

- Tear film hyperosmolarity, immune dysregulation, and neuro-immune interactions are key drivers of Dry Eye Disease (DED) pathogenesis.
- Novel immunotherapies like biologics, stem cell therapy, and exosome-based treatments target the immune system to reduce inflammation and promote ocular surface repair.
- Nanotechnology-based drug delivery systems enhance bioavailability and therapeutic efficacy, allowing for more precise and sustained DED treatments.
- Gene editing technologies, including CRISPR-Cas9, offer potential for modulating immune responses and repairing damaged tissues in DED.
- The ocular surface microbiota influences immune responses and provides new opportunities for personalized immunotherapy in DED management.

## 1 Introduction

Dry Eye Disease (DED) is a common, multifactorial disorder characterized by tear film instability and ocular surface inflammation, along with neurosensory abnormalities. Recent advancements in understanding DED mechanisms have led to new immunotherapeutic strategies, offering fresh hope for clinical treatment. DED can be classified into two main types based on etiology and pathology: aqueous-deficient dry eye and evaporative dry eye. Aqueous-deficient dry eye results from reduced tear production due to lacrimal gland dysfunction, while evaporative dry eye is caused by meibomian gland dysfunction or lipid layer abnormalities, leading to excessive tear evaporation (1). Both types involve loss of tear film homeostasis, leading to symptoms such as ocular discomfort, visual disturbances, tear film instability, hyperosmolarity, ocular surface inflammation, damage, and neurosensory abnormalities.

Epidemiological data shows that DED prevalence varies significantly across regions and populations, with global rates ranging from 5% to 50% and increasing with age (2). The prevalence is notably higher in women, especially postmenopausal women (3). Clinically, DED presents with symptoms such as ocular discomfort (dryness, burning, foreign body sensation), visual disturbances (blurred vision, light sensitivity), tear film instability (uneven tear distribution, reduced tear break-up time), and ocular surface inflammation (conjunctival redness, positive corneal staining) (4).

Current DED treatments include artificial tears to alleviate discomfort and replenish tears, anti-inflammatory medications (cyclosporine A and corticosteroids) to reduce inflammation, punctal plugs to decrease tear drainage, and lifestyle adjustments such as reducing screen time and increasing ambient humidity (5). Despite their efficacy, several challenges remain: individual differences result in varied etiologies and symptoms, affecting treatment outcomes; DED is often chronic, requiring long-term management and follow-up; long-term use of anti-inflammatory drugs can cause side effects; and the lack of standardized diagnostic criteria and sensitive detection methods complicates early diagnosis and precise treatment (6).

In summary, DED is a prevalent and complex ocular surface disease requiring further research and innovative treatments to improve patient quality of life and treatment outcomes. Recent advances, including new biologics, stem cell therapy, gene therapy, and nanotechnology for drug delivery, offer new hope and directions for DED treatment, laying a foundation for future research.

## 2 Pathophysiology of Dry Eye Disease

### 2.1 The role of the immune system in Dry Eye Disease

#### 2.1.1 The role of tear film hyperosmolarity in Dry Eye Disease

**Figure 1:**
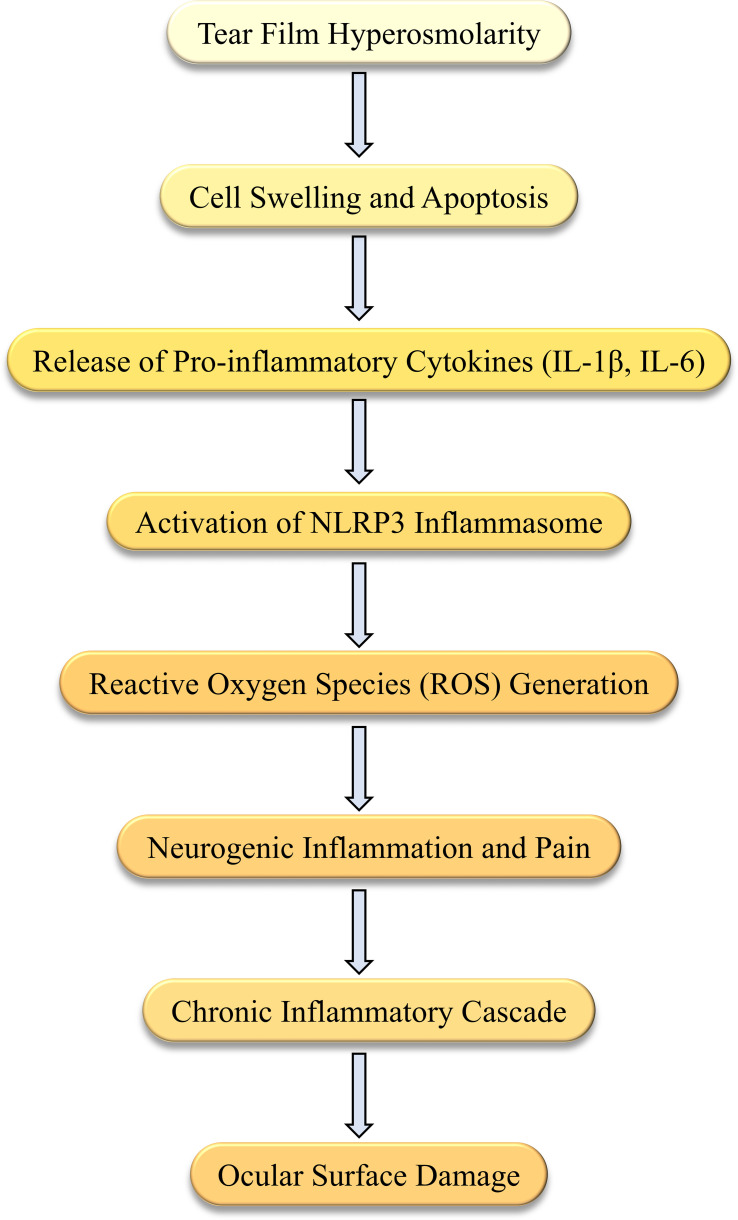
Mechanisms of hyperosmolarity-induced inflammation in Dry Eye Disease (DED). This diagram illustrates the sequence of events triggered by hyperosmolarity in DED. Tear film hyperosmolarity causes cell swelling and apoptosis, leading to the release of pro-inflammatory cytokines, such as IL-1β and IL-6. This activates the NLRP3 inflammasome and generates reactive oxygen species (ROS). The resulting neurogenic inflammation initiates a chronic inflammatory cascade, which further damages the ocular surface.

Tear film hyperosmolarity is a central pathological change in DED, inducing and exacerbating inflammatory responses through various mechanisms. Firstly, a hyperosmolar environment directly damages corneal epithelial cells, causing an imbalance in intracellular and extracellular osmotic pressure, leading to cell swelling and apoptosis. Tear film hyperosmolarity causes morphological changes and apoptosis in corneal and conjunctival cells, which triggers an inflammatory cascade, including the loss of mucin-producing cells, further destabilizing the tear film and perpetuating a vicious cycle of disease (7). Hyperosmotic stress induces the release of a series of pro-inflammatory cytokines and chemokines, such as IL-1β, IL-6, TNF-α, and MMP-9, which attract and activate immune cells, thereby exacerbating the inflammatory response. Studies have shown that hyperosmolarity activates the NLRP3 inflammasome, leading to excessive generation of reactive oxygen species (ROS) in human corneal epithelial cells, triggering inflammatory responses (8). Additionally, hyperosmotic stress activates the nuclear factor kappa B (NF-κB) signaling pathway, increasing the production of inflammatory mediators and further promoting inflammation (9). Hyperosmotic stress also activates autophagy pathways, which regulate cellular metabolism and degradation pathways, reducing inflammation and protecting cell survival. The protective role of autophagy in DED is becoming increasingly recognized. Research indicates that under hyperosmotic stress, autophagy activation can reduce the release of inflammatory mediators, promote cell survival, and protect corneal epithelial cells (10). Hyperosmolarity also affects the nervous system by releasing neuropeptides such as substance P, further exacerbating inflammation and pain. Studies show that in a hyperosmolar environment, corneal nerve sensitivity to cold stimuli is increased, which may be the neural basis for cold-induced discomfort experienced by DED patients (11). Additionally, hyperosmolarity can trigger neurogenic inflammation, further activating immune cells and worsening ocular surface inflammation (12). These findings suggest that hyperosmolarity induces and exacerbates inflammatory responses in DED through multiple mechanisms, providing new perspectives and targets for DED treatment (**Figure 1**).

#### 2.1.2 The role of innate immunity in Dry Eye Disease

The innate immune system plays a crucial role in the onset and progression of DED. As a complex multifactorial condition, DED involves various immune and inflammatory mechanisms. Research indicates that the innate immune system, by recognizing environmental stressors and pathogens, triggers a series of inflammatory responses pivotal in the pathological development of DED (13). Corneal epithelial cells respond to environmental stressors, such as low humidity and hyperosmolarity, by inducing an imbalanced activation of the NLRP3 and NLRP6 inflammasomes through oxidatively damaged mitochondrial DNA. This leads to the activation of caspase-8 and BRCC36, triggering the maturation and secretion of IL-1β and IL-18, thereby exacerbating local inflammation (14). Moreover, epithelial cells and dendritic cells in DED act as environmental sensors, initiating innate immune responses. When exposed to hyperosmolarity and dehydration, these cells activate Toll-like receptors (TLRs) and other pattern recognition receptors (PRRs), leading to innate immune reactions. These reactions not only cause acute inflammation but also guide adaptive immune responses, promoting chronic inflammation (15). Despite understanding the importance of innate immunity in DED, many questions and challenges remain. For example, while it is known that innate immune responses play a key role in the early initiation of DED, it is not fully understood why some patients fail to resolve local inflammation, leading to chronic inflammation. This suggests that the interaction between innate and adaptive immunity is critical in the pathogenesis of DED and requires further investigation (16). Research also indicates that the synergistic action of the NLRP3 and NLRC4 inflammasomes induces inflammatory responses in corneal epithelial cells through a GSDMD-dependent pyroptosis mechanism. TLR4-induced caspase-8 activation plays a crucial role in this inflammatory response. These findings offer new therapeutic targets, suggesting that inhibiting the activity of these inflammasomes could potentially alleviate inflammation in DED patients (17). Future research should explore the dynamic changes in innate immune mechanisms at different stages of DED and their interactions with adaptive immune responses. Additionally, developing new therapeutic strategies by modulating innate immune responses is a promising direction. For instance, drugs based on inflammasome inhibitors could provide new treatment avenues for DED.

#### 2.1.3 The role of adaptive immunity in Dry Eye Disease

Adaptive immunity plays a crucial role in the pathogenesis of DED, particularly through the activation of T cells and the secretion of inflammatory mediators. The main participants of the adaptive immune system involved in DED are T cells, including helper T cells (CD4+ T cells), cytotoxic T cells (CD8+ T cells), and regulatory T cells (Tregs). In DED patients, significant changes occur in the proportion and activity of these T cells, which are closely associated with ocular surface inflammation and tissue damage. Studies have shown that CD4+ T cells, particularly Th1 and Th17 cells, play important roles in the inflammatory response of DED. Th17 cells secrete IL-17, which promotes the release of other inflammatory mediators, exacerbating the inflammatory response. Th1 cells secrete IFN-γ, which also plays a critical role in the inflammatory process (18). CD8+ T cells contribute to ocular surface damage by releasing cytotoxic granules, such as perforin and granzyme, which directly attack epithelial cells, leading to cell damage and death. These cells are significantly increased in the ocular surface tissues of DED patients, further aggravating tissue damage and inflammation (19). Tregs are essential for maintaining immune tolerance and suppressing excessive inflammatory responses. In DED patients, the proportion of Tregs is significantly reduced, leading to uncontrolled inflammation and exacerbation of ocular surface damage (19). Adaptive immune responses are involved in the pathological process of DED through various mechanisms. Antigen-presenting cells (APCs), such as dendritic cells, capture environmental antigens on the ocular surface and present them to T cells via MHC molecules, activating specific T cell responses (20, 21). Activated T cells release a large number of inflammatory mediators, such as IL-17 and IFN-γ, which recruit and activate other immune cells, further exacerbating the inflammatory response and tissue damage (22). Adaptive immune responses can persist and spread through positive feedback mechanisms. For example, IL-17 secreted by Th17 cells can induce the release of more inflammatory mediators, creating a vicious cycle that maintains chronic inflammation (18). These findings highlight the critical role of adaptive immunity, particularly T cell-mediated responses, in the pathogenesis of DED, providing insights into potential therapeutic targets for managing this condition.

#### 2.1.4 Neuro-immune interactions

**Figure 2:**
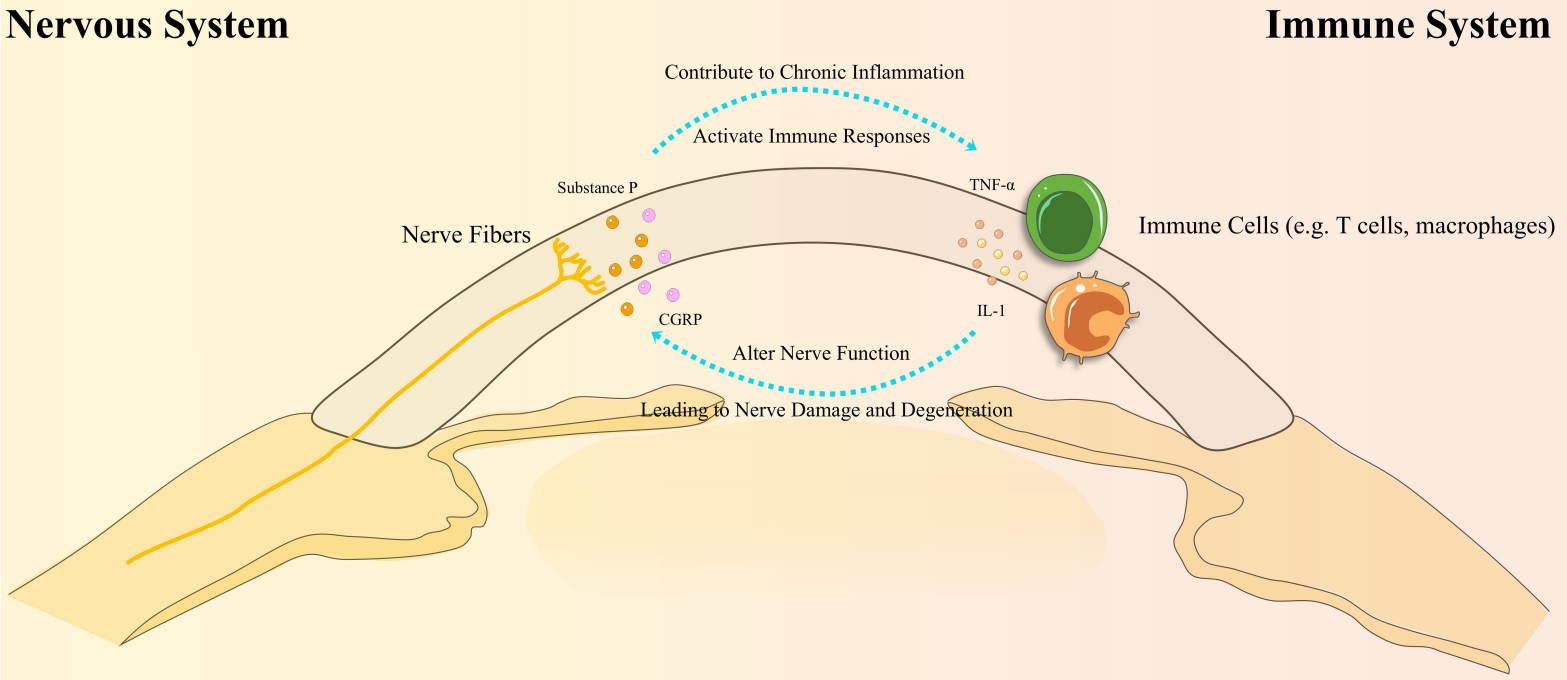
Bidirectional interactions between the nervous and immune systems in Dry Eye Disease (DED). This diagram illustrates the complex bidirectional interactions between the nervous and immune systems in DED. Immune cells, such as T cells and macrophages, release cytokines (e.g., IL-1, TNF-α) that disrupt nerve function, leading to nerve damage and degeneration. In turn, this nerve damage stimulates the release of neuropeptides (e.g., Substance P, CGRP), which activate immune responses and contribute to chronic inflammation. This cycle of inflammation leads to further ocular surface damage, perpetuating the disease process in DED.

The cornea is one of the most densely innervated tissues in the human body, regulated by sensory signals from trigeminal ganglion neurons. Corneal nerves are essential for maintaining ocular surface homeostasis by regulating tear secretion, releasing epithelial trophic factors, and sustaining the blink reflex (23, 24). DED is a multifactorial ocular surface disorder marked by tear film instability and inflammation, often accompanied by corneal nerve degeneration and dysfunction (25). In recent years, the role of neuro-immune interactions in DED pathogenesis has gained significant attention (23, 24). **Figure 2** illustrates the complex bidirectional feedback loop between the nervous and immune systems in DED, showing how nerve dysfunction and immune activation perpetuate ocular surface inflammation. Understanding these interactions offers potential therapeutic targets to disrupt the cycle of chronic inflammation and nerve damage. Further research in this area is crucial for elucidating DED’s pathophysiological mechanisms and developing novel therapeutic strategies.

##### 2.1.4.1 Corneal nerve degeneration and ocular surface inflammation

A key characteristic of DED is corneal nerve degeneration, which is closely linked to ocular surface inflammation (26). In DED patients, corneal nerve damage is common and contributes not only to reduced tear film secretion but also to impaired sensory nerve function, causing pain and discomfort. Animal model studies have shown that desiccating stress and lacrimal gland excision lead to the overexpression of inflammatory cytokines, including IL-1β, IL-6, and TNF-α (27). The persistent presence of these mediators sensitizes and damages corneal sensory nerves, resulting in symptoms such as pain and burning sensations. Substance P (SP) plays a critical role in this process as a pain mediator, and its increased release triggers neural activation, leading to pain and discomfort (28). As inflammation progresses, corneal nerve structures, including intraepithelial nerve terminals and the subbasal nerve plexus, undergo significant damage, reducing corneal sensitivity (29). Research has shown that IL-1β is pivotal in corneal nerve degeneration, and the local application of its antagonist (IL-1Ra) can effectively prevent this degeneration, highlighting IL-1’s role in neuro-immune interactions (30).

##### 2.1.4.2 Role of neuropeptides in neuro-immune interactions

Neuropeptides play a crucial role in the neuro-immune interactions of DED (31). Substance P (SP), a key neuropeptide produced by corneal neurons, is abnormally elevated in DED, inducing neurogenic inflammation and promoting a cascade of ocular surface inflammatory responses (32). SP facilitates the mobilization and maturation of antigen-presenting cells (APCs), driving a Th17 cell response that exacerbates ocular surface inflammation (33). Furthermore, SP promotes the conversion of effector Th17 cells into memory Th17 cells, leading to persistent hyperalgesia and chronic pain (34). Elevated SP levels also increase the proportion of NK1R-positive Tregs, which have impaired regulatory functions, further aggravating inflammation (35). SP also has pro-angiogenic properties, contributing to the formation of new lymphatic vessels in the cornea, thereby worsening the pathological process of DED (36).

Calcitonin gene-related peptide (CGRP) plays a dual role in DED. On one hand, CGRP inhibits inflammatory cell activation and the release of inflammatory mediators, exerting anti-inflammatory effects and promoting wound healing (37). On the other hand, under conditions of nerve injury and inflammation, increased CGRP release can trigger neurogenic inflammation, intensifying discomfort on the ocular surface (38). The complex mechanisms of CGRP’s actions highlight the multiple layers of neuro-immune regulation in the pathogenesis of DED.

##### 2.1.4.3 Regulation of neural function by immune cells

Neuro-immune interactions involve not only the regulation of immune responses by the nervous system but also the influence of immune cells on neural function. Immune cells, particularly T cells, macrophages, and dendritic cells, affect the growth and function of nerve fibers through the secretion of cytokines and chemokines (39, 40). Th17 cells and intraepithelial γδ T cells secrete IL-17, which plays a key role in promoting corneal nerve regeneration and modulating corneal immune responses (41–44). Abnormal activation of these cells can lead to neural dysfunction (45). Additionally, elevated tear osmolarity and the accumulation of inflammatory mediators in DED patients further disrupt nerve and immune cell functions, exacerbating the complexity of the disease (25).

##### 2.1.4.4 Roles of other neuropeptides

In addition to SP and CGRP, other neuropeptides such as α-melanocyte-stimulating hormone (α-MSH) and neuropeptide Y (NPY) also play significant roles in the neuro-immune regulation of DED. α-MSH promotes ocular surface immune tolerance by inhibiting pro-inflammatory cytokine production and promoting the secretion of anti-inflammatory cytokines like IL-10 (46). NPY, through receptor binding, inhibits the production of inflammatory mediators, reduces immune cell activation and infiltration, and aids in neural repair and regeneration, thereby maintaining immune homeostasis on the ocular surface (47). Recent studies have also found that somatostatin (SST), an immunosuppressive peptide produced by TRPV1+ nerves in the cornea, exerts inhibitory effects by binding to receptors on resident immune cells, including macrophages, γδ T cells, mast cells (MCs), and eosinophils (48–50).

In summary, neuro-immune interactions play a pivotal role in the pathogenesis of DED. Neuropeptides such as SP, CGRP, α-MSH, NPY, and SST profoundly influence the inflammatory state and neural function of the ocular surface by regulating immune cell activation, inflammatory mediator release, and neurogenic inflammation. Research into these neuropeptides and their signaling pathways deepens our understanding of DED’s pathophysiological mechanisms and provides potential targets for future immunotherapies. Although significant progress has been made in understanding these interactions, many questions remain. Future research should focus on elucidating the spatiotemporal dynamics of neuro-immune interactions at different stages of DED, as well as clarifying the specific mechanisms of neurotransmitters, neuropeptides, and cytokines. Additionally, large-scale clinical studies are needed to validate the clinical relevance of these findings in the diagnosis and treatment of DED, potentially offering new therapeutic hope for patients.

### 2.2 Pathological mechanisms of Dry Eye Disease

DED involves several pathological mechanisms, including tear film hyperosmolarity, ocular surface inflammation, and neurosensory abnormalities. Hyperosmolar conditions trigger acute immune responses on the ocular surface, which subsequently activate adaptive immune responses, leading to immune system dysregulation. This initiates a vicious cycle, resulting in ocular surface damage. Hyperosmolarity-induced inflammation involves early innate immune responses from epithelial cells and immune cells like macrophages and dendritic cells, followed by chronic inflammation from adaptive immune cells like T cells and B cells (51). The key mechanisms involved include (1): Inflammatory Response: Inflammation is a core mechanism in DED. Environmental stressors like dryness and hyperosmolarity induce inflammatory responses in ocular surface epithelial cells and immune cells, releasing inflammatory mediators and activating adaptive immune responses. Common inflammatory mediators in the tears and ocular surface tissues of DED patients include IL-1β, IL-6, and TNF-α. These factors mediate acute inflammatory responses and lead to chronic inflammation and tissue damage by activating adaptive immune responses (17). (2) Involvement of Immune Cells: Multiple immune cells participate in DED’s inflammatory response. Innate immune cells like neutrophils, macrophages, and dendritic cells play roles in the initial inflammatory response, while adaptive immune cells like T cells and B cells are crucial in chronic inflammation. Th17 cells play a pivotal role in ocular surface inflammation, while a reduction in Tregs exacerbates the inflammatory response. Studies show that DED patients’ ocular surface tissues exhibit a significant increase in Th17 cells and a notable decrease in Tregs, resulting in an imbalance that exacerbates inflammation and tissue damage (52). (3) Role of Cytokines and Chemokines: Various cytokines and chemokines like IL-1β, IL-6, and TNF-α play crucial roles in DED. These mediators drive acute inflammation and lead to chronic inflammation and tissue damage by activating adaptive immune responses. Studies show that these cytokines are significantly elevated in the tears of DED patients and positively correlate with disease severity. These cytokines activate downstream signaling pathways, further exacerbating inflammation and ocular surface damage (53).

Recent studies have highlighted the importance and mechanisms of γδ T cells in DED. Firstly, research found that γδ T cells are a major source of IL-17A in the conjunctiva of mice, and IL-17A plays a crucial role in DED pathogenesis. γδ T cells produce IL-17A, which promotes the disruption of the corneal epithelial barrier, exacerbating ocular surface inflammation (54). In DED patients and experimental mouse models, the proportion of γδ T cells in the conjunctival epithelium is significantly elevated, and these cells secrete IL-17A, leading to the infiltration of inflammatory cells and damage to the ocular surface tissues (54).

Recent research has revealed newly discovered inflammatory pathways involved in DED. For example, the cGAS-STING pathway, which recognizes double-stranded DNA (dsDNA) and initiates inflammatory responses, is a crucial mechanism in DED immune-inflammatory responses (55). Studies have shown that oxidized mitochondrial DNA activates caspase-8 and BRCC36, leading to NLRP3 inflammasome activation and NLRP6 inhibition, further stimulating the maturation and secretion of IL-1β and IL-18 (14). Pyroptosis mediated by NLRP12 and NLRC4 in corneal epithelial cells requires TLR4-induced caspase-8 activation and is accompanied by IL-33 processing (17). Integrative analyses of tear proteomics and metabolomics have uncovered key inflammation-related molecules and regulatory pathways in DED (56).

### 2.3 Summary for DED pathophysiology

**Figure 3:**
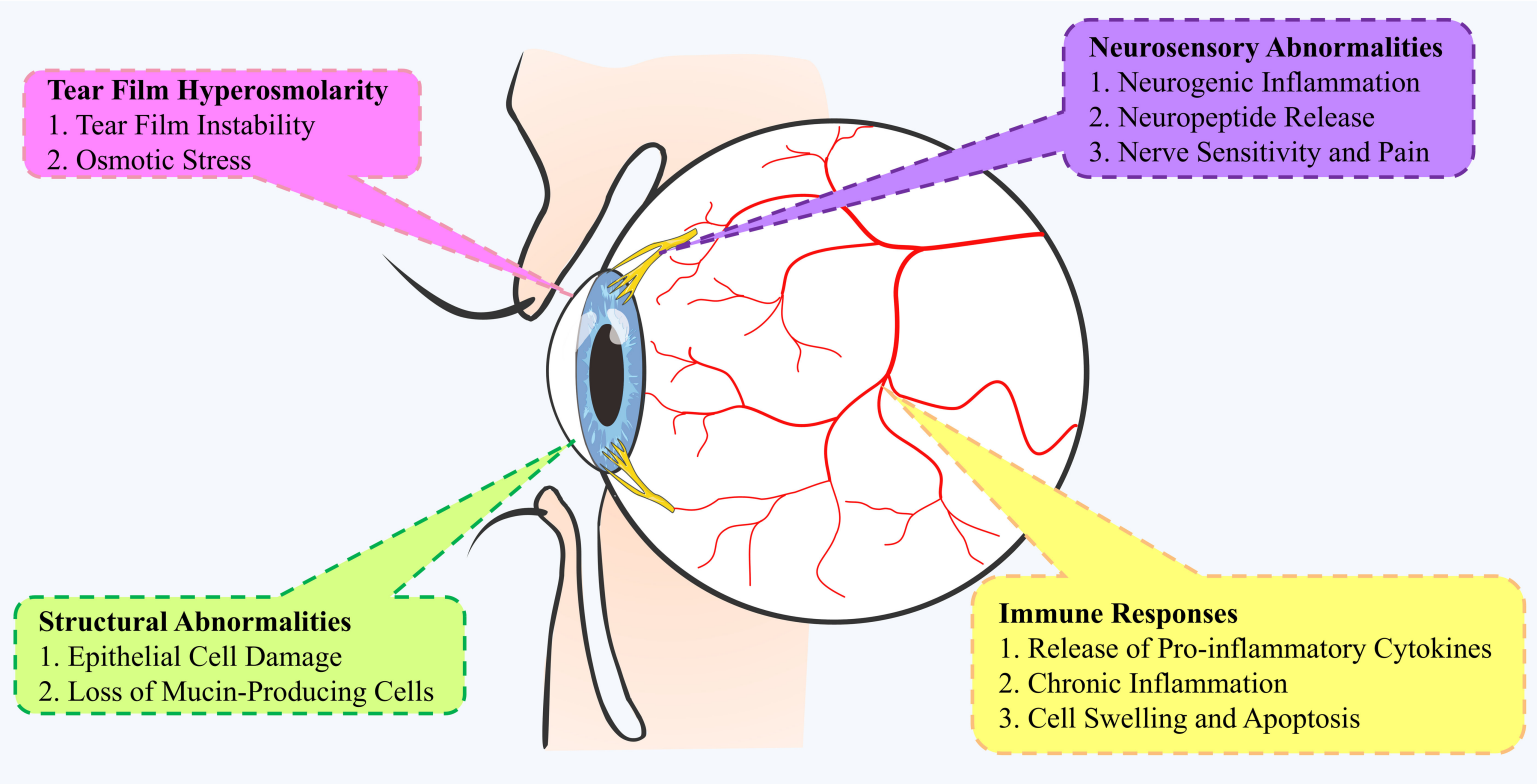
Mechanisms of pathophysiology in the development of Dry Eye Disease (DED). This mind map illustrates the complex mechanisms underlying the pathophysiology of DED. It highlights key pathways, including structural abnormalities (e.g., pro-inflammatory cytokine release, cell swelling), ocular surface inflammation (e.g., epithelial cell damage, loss of mucin-producing cells), and neurological abnormalities (e.g., nerve sensitivity, neuropeptide release). Tear film hyperosmolarity plays a central role, causing tear film instability and osmotic stress. These pathways interact, contributing to the development and progression of DED.

DED involves several pathological mechanisms, including tear film hyperosmolarity, ocular surface inflammation, and neurosensory abnormalities (**Figure 3**). Hyperosmolarity triggers acute immune responses, leading to chronic inflammation mediated by both innate (neutrophils, macrophages, dendritic cells) and adaptive (T cells, B cells) immune cells. Key inflammatory mediators like IL-1β, IL-6, and TNF-α are elevated in DED, contributing to tissue damage. Adaptive immunity, especially T cells, plays a critical role, with Th17 cells exacerbating inflammation and reduced Tregs failing to control it. Neuro-immune interactions further complicate DED, with nerve damage triggering inflammation and immune responses affecting nerve function. Recent discoveries of inflammatory pathways, such as cGAS-STING and NLRP3 inflammasome activation, provide new insights and potential therapeutic targets.

## 3 Traditional immunotherapy for Dry Eye Disease

### 3.1 Anti-inflammatory treatment

Nonsteroidal anti-inflammatory drugs (NSAID: NSAIDs alleviate inflammation by inhibiting cyclooxygenase (COX) enzymes and reducing prostaglandin production. NSAIDs are primarily used to reduce ocular surface inflammation in the treatment of DED. While NSAIDs effectively relieve symptoms, long-term use may cause corneal epithelial damage and other side effects. Recent studies suggest that using functional cationic nanoemulsions (NEs) as drug carriers enhances NSAID bioavailability and extends retention time on the ocular surface, improving therapeutic efficacy (57).

Corticosteroids: Corticosteroids are commonly used anti-inflammatory agents that act by inhibiting inflammatory pathways and are widely utilized in the short-term treatment of DED, particularly in patients who do not respond to conventional therapies (58, 59). Topical corticosteroids, such as 0.5% loteprednol, can rapidly alleviate ocular surface inflammation and improve patient symptoms (32, 60). Additionally, 0.5% loteprednol etabonate has been shown to be effective in treating keratoconjunctivitis sicca without affecting intraocular pressure (61). EYSUVIS (0.25% loteprednol etabonate ophthalmic suspension) has also been FDA-approved for the short-term treatment of DED, utilizing mucus-penetrating particle (MPP) technology to enhance drug delivery, demonstrating a favorable safety and tolerability profile (62). Furthermore, 0.1% fluorometholone has shown significant improvement in ocular surface staining and tear film breakup time in randomized controlled trials (63). Despite their efficacy in short-term DED management, long-term use of corticosteroids may result in adverse effects such as elevated intraocular pressure, cataracts, and infections, thereby necessitating the use of low concentrations and short-duration regimens (25, 59). In Europe, “soft corticosteroids” like 0.335% preservative-free hydrocortisone are commonly used for DED management, showing a favorable balance of efficacy and safety (64).

### 3.2 Immunosuppressants

#### 3.2.1 Cyclosporine A

##### 3.2.1.1 Mechanism of action of CsA

Cyclosporine A (CsA) is a potent immunosuppressant widely used in the treatment of DED. Its primary mechanism of action involves entering T cells and binding to cyclophilin, thereby inhibiting the activity of the calcium-dependent phosphatase, calcineurin. This inhibition blocks the dephosphorylation of nuclear factor of activated T cells 1 (NFATc1), leading to a reduction in interleukin-2 (IL-2) levels and suppression of T cell activation and proliferation. Additionally, CsA reduces interleukin-6 (IL-6) levels, decreases the number of activated T lymphocytes, and lowers the expression of inflammatory and apoptotic markers. It also increases the number of goblet cells in the conjunctiva, contributing to the restoration of conjunctival epithelial homeostasis (65).

##### 3.2.1.2 Clinical evidence for CsA in DED treatment

Several clinical studies have demonstrated the efficacy and safety of CsA in the treatment of DED. De Paiva et al. (2012) reported that 0.05% CsA significantly improves DED symptoms, increases conjunctival goblet cell density, and reduces ocular surface inflammation (65). In a multicenter, randomized, double-blind, placebo-controlled study, Chen et al. (2014) compared the efficacy of 0.05% CsA administered twice daily for 8 weeks with a placebo. The results showed significant improvement in symptoms of dryness, foreign body sensation, corneal staining, and Schirmer test scores at weeks 4 and 8, confirming its efficacy and safety in moderate to severe DED patients (66). Furthermore, Sall et al. (2000) compared the efficacy and safety of 0.05% and 0.1% CsA with a placebo in patients with moderate to severe DED. The study found significant improvements in both corneal staining and Schirmer test values for both concentrations of CsA, with 0.05% CsA also showing notable improvements in subjective symptom measures (67).

##### 3.2.1.3 Formulations of CsA

The 0.05% CsA ophthalmic emulsion (Restasis, Allergan) is the most commonly used formulation and has been approved by the U.S. Food and Drug Administration (FDA) to increase tear production in DED patients. Studies have shown that using 0.05% CsA can significantly improve symptoms of DED, increase conjunctival goblet cell density, and reduce ocular surface inflammation. However, achieving significant therapeutic effects typically requires about 3 months of continuous use (68). For more severe cases of DED, particularly those associated with chronic Graft-Versus-Host Disease (cGVHD), higher concentrations of CsA have been explored. Liu et al. (2023) showed that 0.1% CsA provided more significant improvements in severe DED. Additionally, Cequa (0.09% CsA, Sun Pharmaceuticals), a nanomicellar solution, has been FDA-approved to increase tear production in DED patients, aiming to enhance drug bioavailability while minimizing systemic exposure (69).

##### 3.2.1.4 Development of novel CsA formulations

Traditional CsA formulations are typically oil-based emulsions, such as those containing castor oil, which can prolong the retention time of the drug on the ocular surface. However, these formulations may cause irritation and burning sensations, affecting patient tolerance and compliance (70). To improve patient tolerance, several novel CsA formulations have been developed in recent years. OTX-101 (0.09% CsA) is a water-free nanomicellar solution designed to enhance drug permeability and reduce the incidence of burning sensations, thereby improving patient tolerance (71, 72). Klarity-C (0.1% CsA, Imprimis Pharmaceuticals) is a chondroitin sulfate-based emulsion with anti-inflammatory properties, which can reduce corneal edema and enhance patient comfort. Other delivery methods, such as pH-responsive contact lenses based on cellulose acetate phthalate, have been used to improve CsA delivery and therapeutic effects while minimizing adverse reactions (73). These advanced formulations aim to improve the retention time and permeability of CsA on the ocular surface, optimizing the treatment outcome of DED.

##### 3.2.1.5 Adverse effects and adjunctive therapy of CsA

Common adverse effects of CsA include burning or stinging sensations after instillation, conjunctival hyperemia, discharge, tearing, eye pain, foreign body sensation, visual disturbances, or itching. Since CsA may require up to 3 months to achieve maximal therapeutic efficacy, topical steroids are often used as an induction therapy before long-term CsA use to accelerate symptom relief and improve ocular signs (74).

In summary, Cyclosporine A plays a key role in the management of DED by inhibiting T cell activation and proliferation, thereby reducing ocular surface inflammation. Optimizing the concentration and formulation of CsA is crucial to improving therapeutic outcomes and enhancing patient tolerance. Novel formulations such as water-free nanomicellar solutions and chondroitin sulfate-based emulsions show potential in increasing drug bioavailability and reducing adverse effects. Future research should continue to explore the clinical applications of different CsA concentrations and formulations to provide more effective and tolerable treatment options for patients with DED.

#### 3.2.2 Tacrolimus

Tacrolimus, a macrolide lactone, exhibits potent immunosuppressive properties by inhibiting calcineurin, which is essential for T-cell activation. In DED, tacrolimus reduces immune-mediated inflammation on the ocular surface by inhibiting the production of pro-inflammatory cytokines, including IL-2, IFN-γ, and TNF-α. Studies have confirmed the efficacy of 0.03% tacrolimus eye drops in managing severe DED, particularly in cases unresponsive to traditional therapies. Significant improvements in tear film stability and symptom relief were observed, especially in severe DED related to chronic graft-versus-host disease (cGVHD) (75). Recent advances in drug delivery systems, such as cationic liposomes and gellan gum nanoparticles, have improved ocular penetration and bioavailability, overcoming the poor aqueous solubility and permeability of tacrolimus. Tacrolimus, available in both eye drop and ointment formulations, shows promising potential, particularly in severe conditions such as Sjogren’s syndrome, where combining it with platelet-rich plasma (PRP) enhances efficacy. While generally well-tolerated, common side effects include ocular irritation and, in rare cases, increased risk of infection due to its immunosuppressive effects.

#### 3.2.3 Rituximab

Rituximab is a monoclonal antibody that targets B cells, reducing autoimmune responses by depleting B cells. While rituximab is widely used in other autoimmune diseases, its use in DED is less studied. Current research suggests that rituximab may have potential in treating refractory DED, especially in patients unresponsive to conventional treatments (76).

#### 3.2.4 Other immunosuppressants

In addition to cyclosporine A and tacrolimus, other immunosuppressants like methotrexate, azathioprine, and mycophenolate mofetil have been used to treat DED (76). These drugs reduce ocular surface inflammation and protect ocular structures through various mechanisms. As understanding of the immunopathology of DED advances, more novel immunosuppressants and immunomodulators are being developed. For example, histone deacetylase inhibitors (HDACi) regulate the transcription of immunomodulatory genes and show promising anti-inflammatory effects (77). Studies have demonstrated that HDACi can enhance the function of Tregs and reduce the proliferation of effector T cells. In DED treatment, HDACi reduce ocular surface inflammation markers and improve clinical symptoms.

#### 3.2.5 Risk of Ocular Surface Squamous Neoplasia (OSSN) associated with immunosuppressive therapies in Dry Eye Disease

Ocular Surface Squamous Neoplasia (OSSN) has been reported in patients receiving immunosuppressive treatments for both ocular and systemic diseases, such as cyclosporine A (CsA) and tacrolimus (78, 79). Although OSSN is relatively rare in this context, it warrants careful attention from clinicians. The immunosuppressive effects of these agents, particularly with long-term use, may suppress immune function and increase the risk of tumor development.

Cyclosporine A, tacrolimus, and the risk of OSSN: CsA and tacrolimus are effective immunosuppressants that reduce ocular surface inflammation by inhibiting T-cell activation. CsA directly inhibits T-cell activation, while tacrolimus achieves this through calcineurin inhibition. Both agents are widely used for treating DED and other immune-mediated ocular surface diseases, with tacrolimus being especially effective in severe cases, such as DED associated with chronic graft-versus-host disease (cGVHD).

However, long-term use of these agents has been associated with an increased risk of developing OSSN. Prolonged CsA therapy may weaken immune surveillance on the ocular surface, leading to uncontrolled cell proliferation. This risk may be heightened by tumor-promoting factors such as UV exposure and viral infections, as CsA’s immunosuppressive properties impair normal immune regulation (80). Similarly, tacrolimus, through its calcineurin inhibition, may compromise the ocular surface’s antiviral and antitumor defenses. Case reports suggest an increased incidence of OSSN with long-term tacrolimus use, as its immunosuppressive effects can facilitate OSSN development (81).

Clinical recommendations: Patients undergoing long-term CsA or tacrolimus therapy for DED should be closely monitored, especially those with higher risk factors, such as a history of ocular surface lesions, prolonged UV exposure, or immunocompromised states. Regular ophthalmic evaluations are essential to detect suspicious changes on the ocular surface, such as abnormal growths or ulcerations on the cornea or conjunctiva. If OSSN is suspected, immediate discontinuation of immunosuppressive therapy and further investigation should follow (82).

Conclusion: While CsA and tacrolimus are effective in managing DED, their use may carry a risk of developing OSSN. Clinicians must balance the therapeutic benefits of these agents with the potential risks, considering patient-specific factors. Continuous monitoring of ocular surface health in patients on long-term immunosuppressive therapy is crucial for early detection and management of OSSN. Future research should aim to optimize the efficacy and safety of these treatments, explore new formulations, and develop innovative immunomodulators to address the complexities of DED.

### 3.3 Systemic immunosuppression in Dry Eye Disease

Systemic immunosuppression is rarely used solely for DED due to its potential side effects. However, DED often coexists with systemic autoimmune diseases, such as Sjögren’s syndrome, rheumatoid arthritis, and systemic lupus erythematosus, where systemic immunosuppression is necessary (83–85). In these cases, systemic therapies help manage DED by controlling the underlying inflammation contributing to ocular surface disease.

#### 3.3.1 DED in systemic autoimmune diseases

DED frequently accompanies systemic autoimmune diseases, particularly Sjögren’s syndrome, where immune-mediated damage to exocrine glands leads to tear deficiency (86). Chronic activation of both innate and adaptive immune responses affects systemic organs and induces ocular surface inflammation, worsening DED symptoms. Systemic immunosuppressive therapy is essential in these cases to control the autoimmune process, which indirectly improves ocular surface inflammation and DED symptoms (87). For example, systemic lupus erythematosus and rheumatoid arthritis can cause secondary Sjögren’s syndrome, where DED manifests as part of a broader systemic inflammatory condition (88).

#### 3.3.2 Rituximab and other immunosuppressants in systemic diseases

Rituximab, a monoclonal antibody targeting CD20-positive B cells, is used to treat severe DED in patients with systemic autoimmune diseases like Sjögren’s syndrome and rheumatoid arthritis. It works by depleting B cells, reducing autoantibody production, and modulating inflammatory pathways (89). Rituximab has shown effectiveness in managing systemic Sjögren’s syndrome, which indirectly alleviates DED symptoms (90). However, its use is primarily indicated for systemic disease, with DED improvement being a secondary benefit. Other immunosuppressants, such as mycophenolate mofetil, methotrexate, and azathioprine, are also used in systemic autoimmune diseases with secondary DED (91, 92).

While these agents are effective, they come with significant risks. Rituximab can increase infection risk and cause infusion reactions, with long-term use leading to hypogammaglobulinemia and recurrent infections (93). Similarly, mycophenolate mofetil, methotrexate, and azathioprine can cause systemic side effects, which must be considered when treating DED as part of a broader autoimmune condition.

#### 3.3.3 Side effects and risks of immunosuppressants

Oral immunosuppressants, including rituximab, methotrexate, mycophenolate mofetil, and azathioprine, can induce systemic immunosuppression, increasing the risk of infections, organ toxicity, and malignancies (94). Methotrexate, while effective in controlling systemic inflammation, may lead to hepatotoxicity and bone marrow suppression, requiring regular monitoring (95). Mycophenolate mofetil, commonly used for severe autoimmune diseases, may cause gastrointestinal disturbances, cytopenias, and increased risk of opportunistic infections. The use of systemic immunosuppressants in DED is generally restricted to patients with concomitant systemic autoimmune diseases, where the benefits outweigh the risks (96).

#### 3.3.4 Management strategies in clinical practice

For patients with both DED and systemic autoimmune diseases, treatment must be carefully tailored. Systemic immunosuppressants are used to control systemic disease, which can indirectly improve DED. Agents like rituximab, methotrexate, and mycophenolate mofetil are employed when indicated, while local treatments, such as artificial tears, topical cyclosporine A, and anti-inflammatory agents, are used to manage ocular surface inflammation directly (6).

An integrated approach combining systemic and local therapies can significantly improve patient outcomes, reducing the burden of DED and enhancing quality of life. Regular monitoring is essential to detect side effects early and adjust treatment accordingly (97).

Conclusion: Systemic immunosuppressants play a role in managing DED when it coexists with systemic autoimmune diseases. The primary aim is to control the autoimmune process, which indirectly alleviates DED. Clinicians must weigh the benefits of systemic therapy against potential risks and employ a multidisciplinary approach to optimize care.

## 4 Emerging immunotherapies for Dry Eye Disease

### 4.1 Biologics

Biologics target specific immune pathways to alleviate inflammatory responses in DED. These drugs work by blocking inflammatory mediators, such as cytokines, thereby reducing ocular surface inflammation and immune reactions. Representative biologics include IL-17 inhibitors and TNF-α inhibitors.

#### 4.1.1 IL-17 inhibitors

IL-17, secreted by Th17 cells, plays a significant pro-inflammatory role by promoting the recruitment and activation of inflammatory cells and increasing the release of inflammatory mediators, contributing to DED pathogenesis. IL-17 inhibitors reduce ocular surface inflammation through several mechanisms (1): IL-17 inhibitors decrease the recruitment and activation of inflammatory cells, significantly lowering ocular surface inflammation. Studies have shown that IL-17 inhibitors can notably reduce neutrophil and macrophage recruitment and activation, thus alleviating ocular surface inflammation (98). This mechanism is crucial for mitigating both acute and chronic inflammatory symptoms of DED (2). IL-17 inhibitors modulate inflammatory mediator expression. By inhibiting IL-17 signaling, these inhibitors reduce pro-inflammatory cytokines like IL-1β, TNF-α, and IL-6, which play important roles in DED pathology (3). By reducing inflammation, IL-17 inhibitors significantly improve the integrity and function of the corneal and conjunctival epithelium, enhancing tear film stability and ocular surface health. This helps alleviate DED symptoms and promotes the repair and regeneration of ocular surface tissues. IL-17 inhibitors, such as Secukinumab (a fully human anti-IL-17A monoclonal antibody), effectively reduce inflammation in DED (99, 100).

#### 4.1.2 TNF-α inhibitors

TNF-α is a key pro-inflammatory cytokine involved in various inflammatory diseases. In DED, TNF-α induces inflammatory responses, promotes cell apoptosis, and disrupts tissue structures. TNF-α inhibitors block TNF-α activity, reducing inflammation and alleviating DED symptoms. The main mechanisms include (1): TNF-α inhibitors reduce the production of pro-inflammatory cytokines like IL-6, IL-1β, and IL-17 by blocking TNF-α activity, thus lowering ocular surface inflammation (101). (2) TNF-α inhibitors inhibit immune cell activation, such as T cells and macrophages, reducing their infiltration and inflammatory response on the ocular surface. By inhibiting these immune cells, TNF-α inhibitors effectively decrease ocular surface inflammation. (3) By reducing inflammation, TNF-α inhibitors significantly improve the structure and function of ocular surface tissues, promoting corneal and conjunctival repair and regeneration. This mechanism helps alleviate DED symptoms and improves patient quality of life. TNF-α inhibitors, such as Adalimumab, have shown potential in treating severe DED, especially for patients unresponsive to conventional treatments (62).

#### 4.1.3 Lifitegrast

Lifitegrast is a novel immunomodulator that has garnered significant attention for the treatment of DED (102, 103). Its mechanism of action differs from other immunosuppressants such as cyclosporine A and tacrolimus. Lifitegrast acts as a lymphocyte function-associated antigen-1 (LFA-1) antagonist, inhibiting the binding of LFA-1 to intercellular adhesion molecule-1 (ICAM-1), a key interaction in T-cell activation and migration. By blocking this interaction, lifitegrast reduces T-cell activation and infiltration, thereby diminishing ocular surface inflammation and alleviating DED symptoms (104).

Mechanism of action: In the pathophysiology of DED, T-cell activation and migration are critical in sustaining the inflammatory response. ICAM-1, an adhesion molecule expressed on epithelial and endothelial cells, is significantly upregulated on the ocular surface of DED patients. The binding of ICAM-1 to LFA-1 on T-cells promotes T-cell adhesion, migration, and activation, exacerbating ocular surface inflammation. Lifitegrast competitively inhibits the binding of LFA-1 to ICAM-1, thereby reducing T-cell activation and infiltration. This, in turn, decreases ocular surface inflammation and improves DED symptoms (105).

Clinical efficacy: Several clinical studies have confirmed the efficacy and safety of lifitegrast in treating DED. In Phase III trials, lifitegrast significantly improved both symptoms (e.g., ocular discomfort, foreign body sensation) and signs (e.g., corneal fluorescein staining score) of DED. One study reported a rapid onset of symptom relief within two weeks of initiation, faster than other immunosuppressants like cyclosporine A (106). Additionally, a systematic review and meta-analysis found lifitegrast superior to placebo in improving parameters such as total corneal staining score (TCSS), tear break-up time (TBUT), and ocular surface disease index score (OSDI) (107).

Safety and tolerability: Lifitegrast has been associated with relatively few, typically mild and transient side effects. The most common adverse events include short-term ocular irritation, dysgeusia (altered taste), and transient blurred vision. These side effects are generally mild and resolve shortly after drug administration. Compared to cyclosporine A, which often causes burning and stinging sensations, lifitegrast offers a more tolerable treatment option for patients’ intolerant to other medications (108).

Comparison with other treatments: Lifitegrast’s mechanism of action is distinct from other immunosuppressants such as cyclosporine A and tacrolimus. While cyclosporine A inhibits T-cell activation, lifitegrast blocks the LFA-1/ICAM-1 interaction, preventing T-cell adhesion and migration. This unique mode of action allows lifitegrast to be used in combination with other immunosuppressants, providing a synergistic anti-inflammatory effect, especially in severe or refractory DED cases (109).

In summary, Lifitegrast is a promising therapeutic agent for DED due to its novel mechanism of action, rapid onset of efficacy, and favorable safety profile. It offers an alternative treatment option, particularly for patients who do not respond well to other immunosuppressive agents like cyclosporine A.

#### 4.1.4 Others

The pathophysiological mechanisms of DED involve the dysregulated expression and signaling of various inflammatory mediators and cytokines, with interleukin-1 (IL-1), vascular endothelial growth factor (VEGF), and interferons (IFNs) playing pivotal roles in initiating and sustaining inflammation. Numerous studies have investigated the potential of biologics targeting these inflammatory factors for the treatment of DED, as outlined below.

IL-1 receptor antagonists: Recent studies have elucidated the potential efficacy of IL-1 receptor antagonists in treating DED. IL-1 is a key regulator of inflammation in DED, activating inflammatory pathways by binding to the IL-1R1 receptor, leading to damage to corneal and conjunctival epithelial cells and reduced tear film stability. Elevated IL-1 levels have been observed in the tear fluid and ocular surface tissues of DED patients, directly contributing to ocular surface inflammation and tissue damage (110). IL-1 receptor antagonists block the binding of IL-1α and IL-1β to IL-1R1, inhibiting inflammatory pathways and reducing inflammatory responses. Topical application of IL-1 receptor antagonists has shown promise in alleviating DED symptoms and preventing ocular surface damage (111).

Preclinical studies have demonstrated that local use of IL-1 receptor antagonists reduces ocular surface inflammatory markers, improves corneal epithelial integrity, and enhances tear film stability (112). Clinical trials have also confirmed the efficacy of topical IL-1 receptor antagonists in relieving symptoms, reducing corneal epithelial damage, and improving visual function in DED patients. For example, one study found that the IL-1β inhibitor diacerein reduced ocular surface inflammation and improved DED symptoms (110). Another study revealed that IL-1 receptor antagonists could protect the corneal epithelial barrier by reducing inflammation induced by hyperosmotic stress (113).

In addition to topical application, combining stem cell therapy with IL-1 receptor antagonists is being explored. Research indicates that mesenchymal stem cells alleviate ocular surface inflammation and corneal damage via an IL-1Ra-dependent pathway, offering new prospects for DED treatment (112). These findings strongly support IL-1 receptor antagonists as a promising therapeutic approach for DED, with further research likely to reveal broader clinical applications.

VEGF inhibitors: Recent studies have highlighted the therapeutic potential of vascular endothelial growth factor (VEGF) inhibitors in treating DED by controlling ocular surface inflammation and inhibiting corneal neovascularization (114). VEGF plays a key role in angiogenesis and is closely linked to ocular surface inflammation. In DED patients, elevated VEGF levels are associated with corneal neovascularization and inflammatory responses, which further disrupt the ocular surface’s structure and function, exacerbating symptoms. By inhibiting VEGF, the formation of pathological neovascularization is reduced, mitigating persistent inflammatory damage and providing a dual therapeutic benefit (115).

Although VEGF inhibitors are primarily used to treat retinal diseases such as macular degeneration, recent research suggests their efficacy in controlling corneal neovascularization and inflammation in DED. Studies have shown that anti-VEGF therapy reduces corneal angiogenesis and inflammatory infiltration, alleviating ocular surface symptoms (114). Animal models have demonstrated that topical administration of VEGF inhibitors effectively inhibits corneal neovascularization (116). Ongoing research, including clinical trials, is exploring innovative strategies for anti-VEGF therapy in DED. One study utilized nanotechnology-based carbon dots (C-dots) as carriers for delivering anti-VEGF agents to the ocular surface, enhancing therapeutic efficacy while minimizing side effects (116).

By inhibiting VEGF, pathological neovascularization is reduced, and inflammation-induced damage to the ocular surface is alleviated. Local application of anti-VEGF agents such as bevacizumab and aflibercept has shown efficacy in reducing both inflammation and angiogenesis (116). This dual effect may help restore the normal structure and function of the ocular surface, improving DED symptoms.

In summary, VEGF inhibitors offer a promising dual therapeutic approach in DED by inhibiting corneal neovascularization and reducing ocular surface inflammation. However, further clinical trials and research are necessary to confirm their efficacy and safety.

Interferon therapy: Recent studies have demonstrated that interferons (IFNs), particularly interferon-alpha (IFN-α) and interferon-gamma (IFN-γ), play crucial roles in the immune regulation and anti-inflammatory processes of DED. IFN-γ is a key mediator of Th1 cell-mediated immune responses, contributing to the chronic inflammation in DED. Overexpression of IFN-γ exacerbates ocular surface inflammation and leads to epithelial cell damage (117). IFN-γ antagonists can reduce ocular surface inflammation by inhibiting Th1-mediated immune responses, thus decreasing the infiltration of inflammatory cells and the release of inflammatory mediators (118).

Conversely, IFN-α has been studied for its potential to improve the immune status of the ocular surface and promote corneal epithelial healing. By modulating immune cell activity and reducing inflammatory factor production, IFN-α aids in the repair and regeneration of corneal tissues, alleviating symptoms and ocular surface damage in DED (119). Its therapeutic effect is primarily due to its ability to enhance the anti-inflammatory environment and support tissue healing on the ocular surface.

The dual role of interferon therapy in immune regulation makes it a promising therapeutic strategy for DED. IFN-γ antagonists inhibit harmful Th1-mediated immune responses, while IFN-α promotes tissue repair and maintains immune balance on the ocular surface.

**Figure 4:**
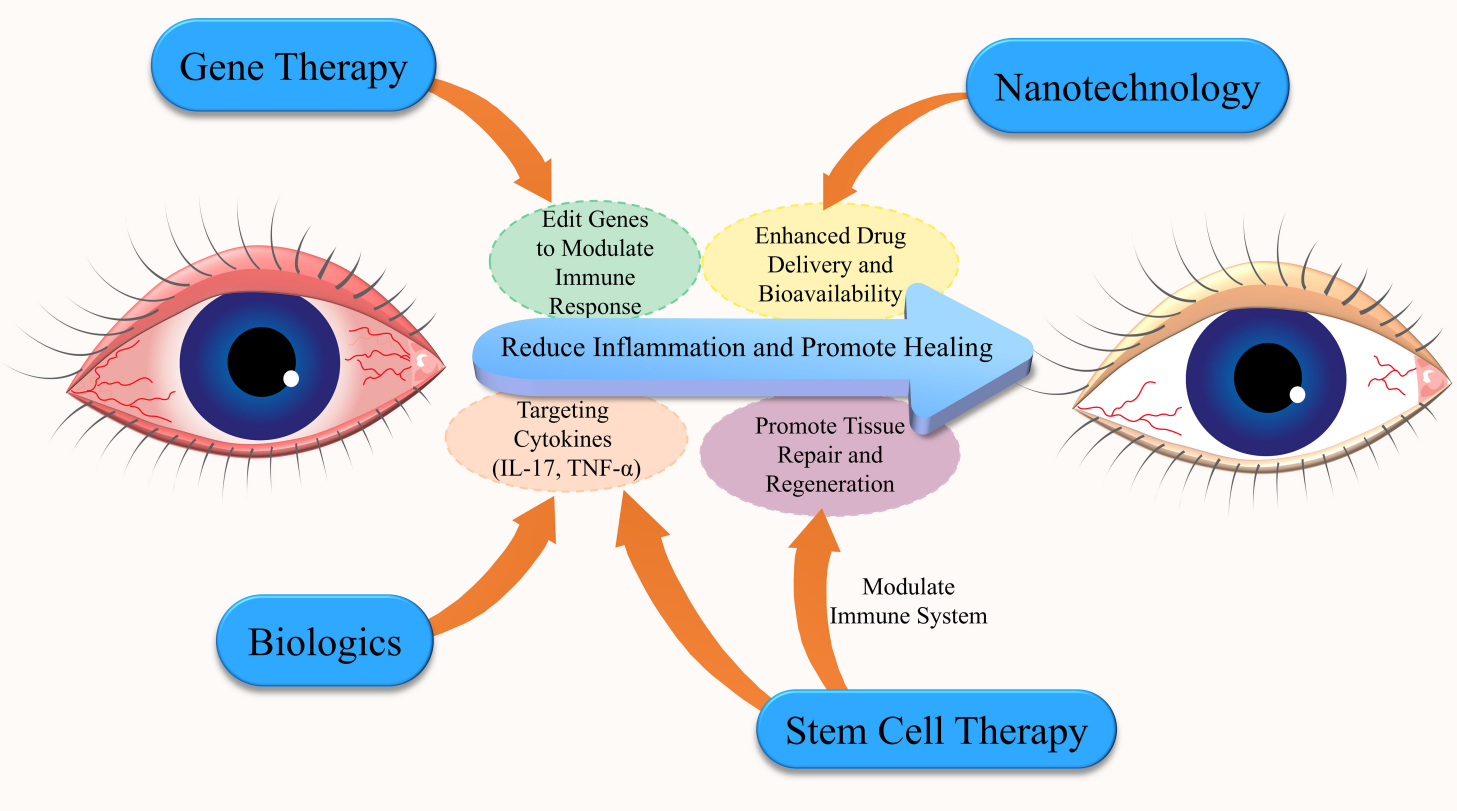
Interconnected emerging immunotherapies for Dry Eye Disease (DED). This diagram highlights the key emerging immunotherapies for DED, emphasizing the interconnected roles of gene therapy, nanotechnology, biologics, and stem cell therapy. Biologics target specific cytokines to reduce inflammation, while stem cell therapy promotes tissue repair and immune modulation. Nanotechnology improves drug delivery and bioavailability, enabling more targeted treatments. Gene therapy involves gene editing to modulate immune responses, reducing inflammation and enhancing healing. These innovative approaches offer a comprehensive strategy for managing DED.

In conclusion, emerging immunotherapies for DED target multiple pathways to prevent and manage the progression of the condition, offering novel treatments, particularly for patients unresponsive to traditional therapies. These therapies—including biologics like IL-1 receptor antagonists, VEGF inhibitors, and interferon therapy—focus on modulating key inflammatory mediators, reducing immune cell activation, and restoring ocular surface integrity (**Figure 4**). As research advances, these innovative approaches show great potential for more effective and targeted DED management.

### 4.2 Stem cell therapy

#### 4.2.1 Tregs types of stem cells

The application of stem cell therapy in DED primarily focuses on mesenchymal stem cells (MSCs). MSCs possess immunomodulatory and regenerative capabilities, promoting the repair and regeneration of ocular surface tissues by secreting various growth factors and cytokines. Studies have shown that MSCs can significantly improve DED symptoms by reducing inflammation, promoting tissue repair, and restoring lacrimal gland function. MSCs and their derivatives represent a non-immunogenic stem cell source with extensive regenerative and anti-inflammatory properties, making them promising candidates for treating DED and other ocular surface diseases (120).

#### 4.2.2 Mechanisms of action and clinical research progress

**Figure 5:**
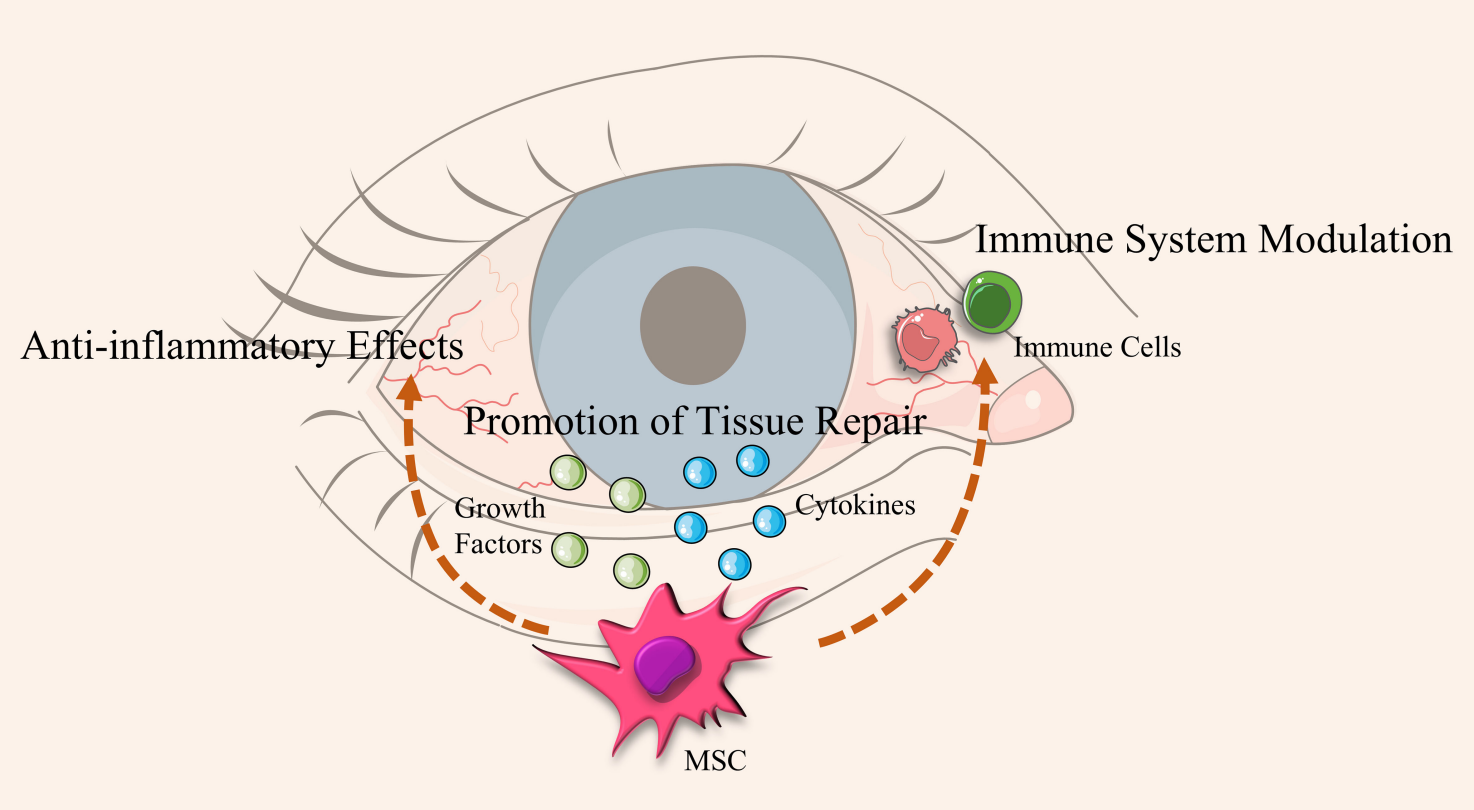
Mechanisms of action of Mesenchymal Stem Cells (MSCs) in alleviating Dry Eye Disease (DED). This diagram illustrates the process by which Mesenchymal Stem Cells (MSCs) alleviate Dry Eye Disease (DED). MSCs secrete growth factors and cytokines that provide anti-inflammatory effects, promote tissue repair, and modulate the immune system. These actions aid in restoring lacrimal gland function and enhancing tear secretion, leading to an overall improvement in ocular surface health.

MSC therapy works through multiple mechanisms, including immunomodulation, anti-inflammatory effects, and tissue regeneration (**Figure 5**). MSCs can alleviate ocular surface inflammation by secreting anti-inflammatory cytokines and inhibiting inflammatory cell activity. Additionally, MSCs promote the repair and regeneration of corneal and conjunctival epithelial cells by secreting various growth factors. In mouse models of DED, MSCs significantly reduced ocular surface inflammation by inhibiting the expression of autophagy markers and inflammatory mediators (121). Studies have shown that MSCs can improve DED symptoms by reducing inflammation, promoting tissue repair, and restoring lacrimal gland function (122). Clinical studies have demonstrated that MSC therapy effectively improves DED symptoms and restores ocular surface health without significant side effects (123).

Overall, MSC therapy shows significant efficacy in improving DED symptoms and restoring ocular surface health without severe side effects. These findings suggest that MSCs and their derivatives have broad application prospects in treating DED.

### 4.3 Gene therapy

**Figure 6:**
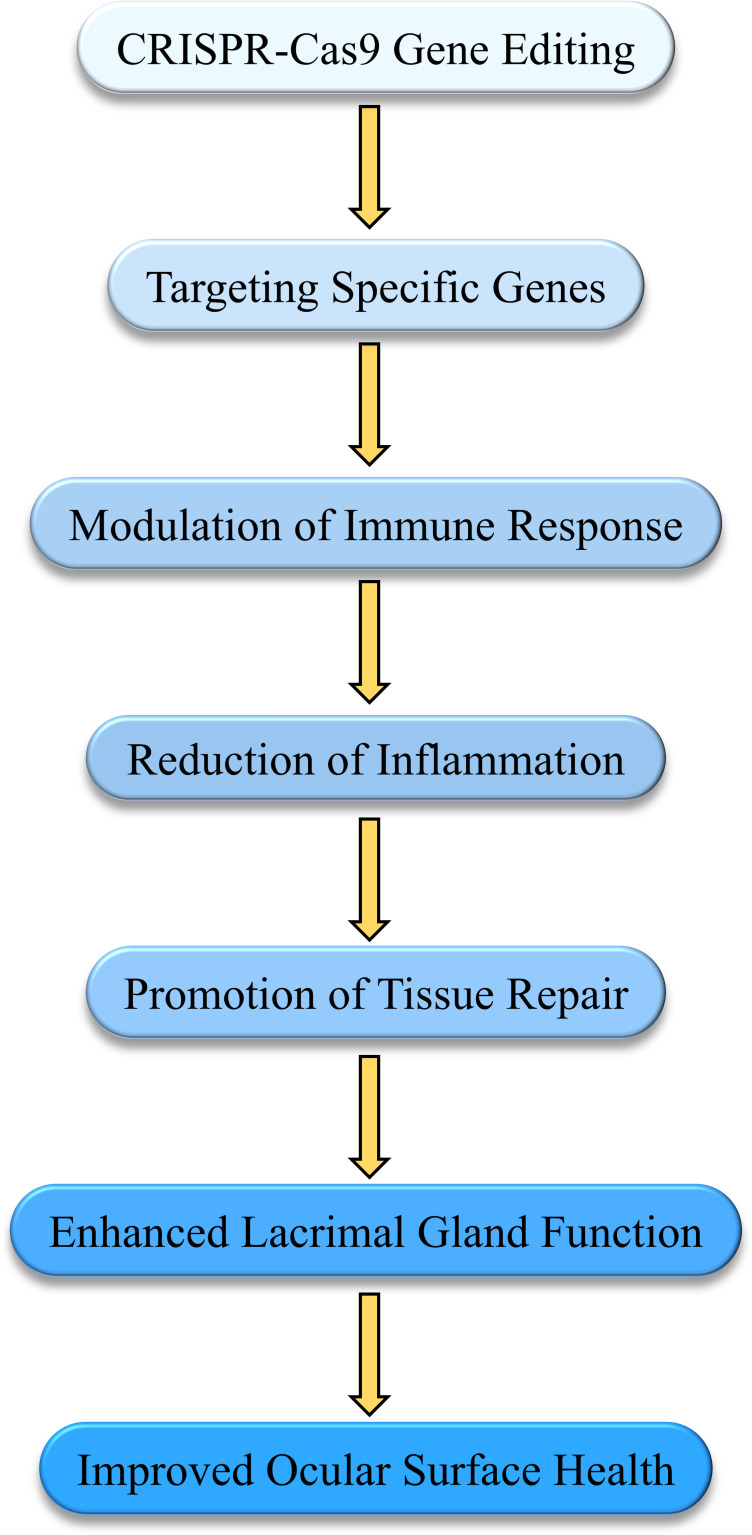
Mechanisms of gene therapy in modulating immune response and tissue repair in Dry Eye Disease (DED). This diagram illustrates how gene-editing technologies, such as CRISPR-Cas9, are applied in the treatment of DED. By targeting specific genes, the therapy modulates the immune response, reducing inflammation and promoting tissue repair. This process enhances lacrimal gland function, leading to improved ocular surface health and alleviation of DED symptoms.

Gene therapy treats diseases by repairing or replacing defective genes. Recently, gene editing technologies like CRISPR-Cas9 have been applied to DED primarily to modulate immune responses and repair damaged tissues. CRISPR-Cas9 can specifically regulate genes associated with DED, reducing inflammation and promoting tissue repair. Studies have demonstrated that CRISPR-Cas9 successfully restored visual function in a mouse model of retinitis pigmentosa, indicating its efficacy in correcting mutation-induced ocular diseases (124). Additionally, using CRISPR-Cas9 to correct pathogenic mutations in mouse models has improved the pathological conditions of retinal diseases, providing a theoretical basis for using CRISPR-Cas9 in treating DED and other ocular diseases (125). Gene editing can specifically regulate genes associated with DED, reducing inflammation and promoting tissue repair (126). The **Figure 6** illustrates how gene-editing technology, such as CRISPR-Cas9, modulates immune response to reduce inflammation and enhance lacrimal gland function, ultimately improving ocular surface health.

Although gene therapy for DED is still in early research stages, preliminary studies suggest its potential in modulating immune responses and promoting tissue repair. A review of CRISPR-Cas9 progress in treating hereditary retinal diseases highlighted its application prospects in DED and other ocular disease (127). Future research must further validate its safety and efficacy. Recent studies summarized CRISPR-Cas9’s advancements in treating various ocular diseases, noting that despite its revolutionary potential, clinical application faces challenges such as off-target effects and long-term stability of gene editing (128).

Gene therapy holds promise as a curative treatment for DED. With ongoing development and optimization, CRISPR-Cas9 gene editing will play an increasingly important role in modulating immune responses and repairing damaged tissues, offering a potential curative approach for DED (129).

### 4.4 Nanotechnology and targeted delivery systems

#### 4.4.1 Application of nanotechnology in drug delivery systems

In recent years, nanotechnology has significantly enhanced drug bioavailability and therapeutic efficacy in drug delivery systems. Nanoparticles as drug carriers can increase drug residence time on the ocular surface, improve stability, and enhance bioavailability, thereby improving therapeutic outcomes. Nanotechnology provides new opportunities to overcome the physiological and biological barriers of the eye. Various nano-drug formulations, including nanomicelles, liposomes, nanoparticles, dendrimers, and nanogels, can reduce drug degradation and increase residence time and bioavailability in ocular tissues.

#### 4.4.2 Mechanisms of action and clinical research progress

Nanotechnology in DED treatment extends beyond enhancing drug stability and bioavailability. It includes using targeted delivery systems to specifically deliver drugs to diseased areas, maximizing therapeutic efficacy while minimizing side effects on healthy tissues. Targeted therapy can significantly improve drug efficacy by delivering drugs specifically to the affected area. For example, nanotechnology in DED drug delivery improves the delivery efficiency of anti-inflammatory and antioxidant drugs (130). Additionally, nano-based drug delivery systems can overcome the eye’s physiological barriers, thereby enhancing drug effectiveness (131).

Nanotechnology also provides sustained and targeted delivery systems. For instance, nanoparticles from human umbilical cord mesenchymal stem cells targeting the IRAK1/TAB2/NF-κB pathway significantly reduced inflammation in a mouse model of DED, increasing tear secretion and corneal integrity (132). Furthermore, nanotechnology-developed biomaterials, such as nano-systems and hydrogels, along with cell and tissue engineering therapies, show great potential in DED treatment (131).

#### 4.4.3 Clinical challenges and future prospects

Despite nanotechnology’s broad prospects in DED treatment, challenges remain, such as achieving efficient targeted delivery, improving drug stability, and enhancing biocompatibility. Future research needs to optimize these delivery systems further to improve therapeutic outcomes and safety. Researchers suggest more clinical trials are needed to verify nanotechnology’s effects in DED treatment, ensuring efficient targeted delivery, improved drug stability, and biocompatibility, as well as overall safety and efficacy (133, 134).

### 4.5 Exosome-based therapies

Exosomes and extracellular vesicles (EVs) are small vesicles secreted by cells that carry various bioactive molecules, including proteins, nucleic acids, and lipids, and play a crucial role in intercellular communication. In recent years, EVs have garnered increasing attention in the treatment of DED due to their capacity to modulate immune responses and promote tissue repair.

#### 4.5.1 Mesenchymal stem cells (MSCs)-derived exosomes

MSC derivatives, such as exosomes and extracellular vesicles (EVs), exhibit significant therapeutic potential in DED. Research indicates that exosomes derived from human umbilical cord MSCs can target the IRAK1/TAB2/NF-κB signaling pathway, significantly reducing inflammation, increasing tear secretion, and enhancing corneal integrity in a mouse model of DED (132). Additionally, MSC-derived EVs can reduce DED severity by inhibiting dendritic cell (DC) function and decreasing the frequency of Th17 cells (135). Another study showed that conditioned medium from human adipose-derived MSCs (hAdMSC-CM) effectively treated DED by improving corneal barrier function and inhibiting benzalkonium chloride (BAC)-induced cytotoxicity and inflammation (136).

#### 4.5.2 iPSCs and their derived MSC exosomes

In recent years, the therapeutic potential of induced pluripotent stem cells (iPSCs) and their derived MSC exosomes in treating DED has gained significant attention. iPSCs possess the ability for unlimited self-renewal and multilineage differentiation, providing a plentiful cell source for DED therapy. Studies have shown that iPSC-derived exosomes exhibit comparable, if not enhanced, immunomodulatory and tissue repair functions compared to MSC exosomes. These exosomes contain various anti-inflammatory factors and growth factors that modulate immune cell activity and promote corneal epithelial cell regeneration, thereby alleviating DED symptoms (137).

Beyond corneal epithelial regeneration, iPSC-derived MSC exosomes demonstrate potential in reducing inflammation. They inhibit the activation of the NLRP3 inflammasome, a key player in ocular surface inflammation, which leads to a reduction in inflammatory cytokines and less ocular surface damage (138). Additionally, iPSC-derived MSC exosomes offer a novel long-term treatment strategy for DED. These exosomes modulate the immune response and promote tissue repair without requiring direct cell transplantation, thus reducing risks of immune rejection and tumorigenicity. Their low immunogenicity and greater stability compared to live cell injections further enhance their safety profile (139).

Overall, iPSC-derived MSC exosomes present a promising approach for DED treatment, offering dual benefits of immune modulation and tissue repair. Continued research into their mechanisms and clinical applications will further strengthen their potential as a therapeutic modality for DED.

#### 4.5.3 Exosomes as delivery vectors for gene therapy

As discussed in Section 4.4, gene therapies like CRISPR-Cas9 hold potential in modulating the immune response and promoting tissue repair in DED. However, the efficacy and safety of gene therapy largely depend on the efficiency and specificity of gene delivery. Exosomes, as natural nanoscale delivery vectors, offer excellent biocompatibility and low immunogenicity, making them suitable for delivering gene-editing tools such as siRNA or Cas9 to precisely target ocular surface tissues. For example, engineered exosomes can be loaded with the CRISPR-Cas9 system or anti-inflammatory genes, allowing for specific regulation of ocular surface cells. Utilizing exosomes for gene delivery not only enhances the efficiency of gene therapy but also reduces potential immune reactions associated with viral vectors (140).

Exosomes have also shown higher specificity and efficiency in gene delivery. For instance, MSC-derived exosome vectors engineered for high expression of CXCR4 have been used for targeted siRNA gene therapy, demonstrating enhanced specificity and efficiency in gene delivery (141). Additionally, exosomes can carry CRISPR-Cas9 ribonucleoprotein complexes for tissue-specific gene therapy, further illustrating their broad applicability in gene therapy (142).

In summary, exosomes, particularly those derived from MSCs and iPSCs, exhibit multiple functions in DED treatment, including immunomodulation, tissue repair, and serving as vectors for gene therapy. By modulating the immune microenvironment of the ocular surface and promoting corneal epithelial cell regeneration, exosomes can alleviate DED symptoms and improve patient quality of life. The combination of exosomes and gene therapy offers new prospects for precision treatment of DED. Future research should further explore the mechanisms and clinical application strategies of exosomes in different DED subtypes to achieve more individualized and effective treatments. The **Table 1** summarizes the current therapeutic options for DED, highlighting each treatment’s potential, mechanism, and safety profile. It is evident that although many therapies are available, each has distinct advantages and limitations. Future treatments, especially those combining advanced gene therapy with nanotechnology-based delivery systems, hold the potential to provide more effective and safer options.

**Table 1:** Treatment summary table for Dry Eye Disease (DED).

Treatment Type	Mechanism of Action	Effectiveness	Potential Side Effects
NSAIDs	Inhibit COX enzymes to reduce inflammation	Moderate efficacy in reducing ocular surface inflammation	Corneal epithelial damage with long-term use
Corticosteroids	Suppress inflammatory pathways	Highly effective for short-term inflammation control	Elevated intraocular pressure, cataracts with long-term use
Cyclosporine A	Inhibits T-cell activation and cytokine release	Effective for moderate to severe DED; increases tear production	Burning or stinging sensation
Tacrolimus	Inhibits T-cell activation via calcineurin blockade	Highly effective, especially in severe cases	Risk of Ocular Surface Squamous Neoplasia (OSSN) with prolonged use
Lifitegrast	Inhibits LFA-1/ICAM-1 interaction to reduce T-cell activation	Rapid symptom relief within 2 weeks	Ocular irritation, dysgeusia
IL-17 Inhibitors	Reduce IL-17 mediated inflammation	Promising in reducing ocular surface inflammation	Limited data on long-term safety
TNF-α Inhibitors	Block TNF-α activity to reduce inflammation	Effective in severe and refractory DED	Risk of infection, systemic effects
Stem Cell Therapy	MSCs reduce inflammation and promote tissue repair	Significant improvement in DED symptoms	Limited data on long-term safety
Gene Therapy (CRISPR-Cas9)	Modulates immune responses, repairs damaged tissues	Experimental, shows potential in preclinical studies	Safety concerns, off-target effects
Nanotechnology	Enhances drug bioavailability and targeted delivery	Improved therapeutic outcomes, prolonged drug action	Biocompatibility and stability challenges
Exosome-Based Therapies	Modulate immune responses, promote tissue repair	Potential for targeted immunomodulation	Limited data, more research needed

## 5 Immunoregulatory strategies for Dry Eye Disease

### 5.1 Role of regulatory T cells (Tregs) in Dry Eye Disease

DED is a multifactorial condition characterized by ocular discomfort, visual disturbances, and tear film instability. Recent studies have highlighted the critical role of Tregs in DED, revealing their impact on disease pathogenesis and potential therapeutic significance.

Mechanisms of Action: Tregs play a pivotal role in maintaining immune tolerance and preventing autoimmune reactions. In DED, Treg dysfunction is considered a key factor contributing to chronic ocular surface inflammation. Research has found that both the number and function of Tregs are reduced in DED patients, leading to exacerbated inflammatory responses. Tregs exert their immunoregulatory effects by secreting anti-inflammatory cytokines and inhibiting inflammatory cell activity. In DED models, antagonizing the neurokinin-1 receptor (NK-1R) can restore Treg function and reduce disease severity (35). Moreover, pigment epithelium-derived factor (PEDF), a broadly expressed anti-inflammatory glycoprotein, can inhibit the maturation of antigen-presenting cells, thereby enhancing Treg function. PEDF prevents the loss of Treg numbers and functionality induced by Th17 cell-related pro-inflammatory cytokines, maintaining Treg levels and reducing disease severity in DED models (143).

Clinical Research and Application Prospects: Recent research indicates that enhancing Treg function or numbers can significantly improve DED symptoms. For instance, PEDF not only enhances Treg immunosuppressive function but also increases Treg numbers and functionality through systemic treatment, thereby reducing ocular surface inflammation and DED symptoms (143). Additionally, blocking NK-1R can restore Treg function and alleviate DED inflammation and symptoms. This approach reduces the effects of the pro-inflammatory neuropeptide substance P (SP) in DED, effectively restoring Treg numbers and suppressive function, and significantly decreasing Th17 cell pathogenic responses, thereby improving DED symptoms (35). In the future, Tregs could become a novel therapeutic target for DED. Enhancing Treg function or numbers may better control ocular surface inflammation and improve patient quality of life. Ongoing clinical trials and studies will further elucidate the potential and application prospects of Tregs in DED treatment, providing more effective and personalized therapeutic options.

In the future, Tregs could become a novel therapeutic target for DED. Enhancing Treg function or numbers may better control ocular surface inflammation and improve patient quality of life. Ongoing clinical trials and studies will further elucidate the potential and application prospects of Tregs in DED treatment, offering more effective and personalized therapeutic options.

### 5.2 Neuropeptides, neurotransmitters, and Dry Eye Disease

As previously discussed, neuro-immune interactions play a significant role in the onset and progression of DED through neuropeptides and neurotransmitters. Antagonists and agonists of these molecules have shown potential therapeutic value in treating DED. Substance P, a neuropeptide associated with various inflammatory states, promotes antigen-presenting cell maturation and enhances inflammatory responses in DED. The NK-1R antagonist, targeting the receptor for substance P, can effectively restore regulatory T cell (Treg) function and inhibit the pathogenic response of Th17 cells, significantly alleviating DED symptoms (35). Diquafosol, a P2Y2 receptor agonist, activates P2Y2 receptors on the ocular surface, increasing tear secretion and tear film stability. Clinical trials have shown that diquafosol significantly improves corneal and conjunctival staining scores, tear film break-up time, and Schirmer test scores in DED patients (144). Pituitary adenylate cyclase-activating polypeptide (PACAP) stimulates tear secretion and inhibits corneal damage. Studies have demonstrated that PACAP significantly enhances tear secretion and reduces corneal damage, improving DED symptoms (145). The TRPM8 receptor, typically associated with cold sensation, can be selectively activated by the TRPM8 agonist Cryosim-3, significantly increasing tear secretion and alleviating DED discomfort (146).

Antagonists and agonists of neuropeptides and neurotransmitters exhibit great potential in treating DED. These drugs can modulate neuro-immune interactions, reduce inflammation, and significantly improve symptoms in DED patients. Future research should continue to explore the clinical applications and efficacy of these treatments.

### 5.3 Microbiome and Dry Eye Disease

#### 5.3.1 Role of ocular surface microbiota

The ocular surface microbiota plays a crucial role in maintaining ocular surface health and immune balance (147–149). Studies have shown significant differences between the ocular surface microbiota of DED patients and healthy individuals. In DED patients, microbial diversity is significantly reduced, and the relative abundance of certain pathogenic bacteria increases. This microbial imbalance may be a key factor in chronic ocular surface inflammation. For instance, research has found that the microbiome of DED patients during eye closure differs significantly from that of healthy individuals, maintaining unique characteristics even after daily saline rinses (150, 151). Additionally, studies have indicated changes in the composition and function of the ocular surface microbiome in DED patients, with notable differences between those with autoimmune-related DED and those with non-autoimmune DED (152). A pilot study using 16S rRNA gene sequencing to analyze the conjunctival bacterial communities of DED patients and healthy controls found a significant increase in Firmicutes bacteria in the conjunctival microbiota of DED patients (152). These findings suggest that the imbalance of the ocular surface microbiota may play a critical role in the pathogenesis of DED.

#### 5.3.2 Potential of treating Dry Eye Disease by modulating the microbiome

Research on treating DED by modulating the microbiome is gradually expanding. Recent studies suggest that specific probiotics can significantly improve DED symptoms. For instance, a study found that a mixture of five probiotics, IRT5, can inhibit the onset of autoimmune DED, increase tear secretion, and reduce corneal damage (153). Additionally, dysbiosis of the gut microbiota may influence systemic immune responses, subsequently affecting ocular surface inflammation (154). Restoring the balance of gut microbiota through methods such as fecal microbiota transplantation or probiotics may offer a new approach to treating DED (155). A study investigating the relationship between anxiety and gut dysbiosis in primary Sjögren’s syndrome (pSS)-related DED patients found a bidirectional relationship, with gut microbiome changes closely related to the severity of DED (156). These studies suggest that modulating the microbiome, particularly the gut and ocular surface microbiomes, could provide personalized and effective treatment options for DED.

### 5.4 Alternative drug delivery methods

While topical eye drops remain the primary treatment modality for DED, alternative drug delivery methods such as subconjunctival injections, implants, iontophoresis, and microneedle delivery have garnered increasing attention in recent years. These novel methods aim to enhance drug bioavailability, extend the duration of therapeutic effects, and minimize local discomfort, offering more effective and better-tolerated treatment options for DED (157).

#### 5.4.1 Subconjunctival injection

Subconjunctival injection involves the direct administration of medication into the subconjunctival tissue, achieving higher local drug concentrations and reducing systemic side effects. For DED, especially in severe or refractory cases, this approach offers an effective therapeutic strategy (158). For instance, anti-inflammatory drugs like corticosteroids and immunosuppressants can be delivered via subconjunctival injection to act directly on the ocular surface and surrounding tissues, thereby mitigating inflammation and alleviating symptoms (159). However, potential adverse effects such as elevated intraocular pressure, infection, and injection site irritation necessitate cautious use in specific clinical contexts.

#### 5.4.2 Implants

Ocular implants provide a sustained drug delivery mechanism, reducing the need for frequent administration. In DED management, implants can encapsulate anti-inflammatory agents, immunomodulators, or lubricants, slowly releasing them to maintain a therapeutic concentration on the ocular surface over time. For example, cyclosporine A micro-implants have been investigated for the long-term control of ocular surface inflammation and reduction of dry eye symptoms. These implants, typically placed in the lacrimal punctum or conjunctival sac, can deliver medication for several months, thus improving patient adherence and providing a more stable therapeutic effect (160, 161). However, the use of implants may be associated with potential side effects such as foreign body sensation, implant site inflammation, or displacement, requiring careful patient selection and monitoring.

#### 5.4.3 Iontophoresis

Iontophoresis is a non-invasive drug delivery technique that uses a mild electric current to facilitate the penetration of drugs across the ocular surface barriers (162, 163). By applying a low electrical current to the eye, drug molecules can more effectively traverse the corneal and conjunctival barriers, reaching deeper ocular tissues. This method has been studied for delivering anti-inflammatory drugs and immunosuppressants like cyclosporine A to the ocular anterior segment, enhancing efficacy and reducing local irritation. Studies have demonstrated that iontophoresis can increase cyclosporine concentration in the cornea, aiding in the more effective alleviation of ocular surface inflammation. Compared to traditional eye drops, iontophoresis offers higher drug bioavailability and less frequent dosing, although further research is needed to validate its long-term efficacy and safety.

#### 5.4.4 Microneedle delivery

Microneedle technology is a minimally invasive method that penetrates the corneal or conjunctival epithelium, delivering drugs directly to the target tissues underlying the ocular surface. This approach can overcome physiological barriers of the ocular surface, enhance drug permeability, and reduce the rapid clearance of drugs in the tear film. For DED, microneedles can be used to deliver anti-inflammatory agents, growth factors, or immunomodulators for a more direct and effective treatment (164). This technique has shown advantages in being minimally invasive, providing controlled release, and achieving efficient drug delivery. However, it is still under investigation, and its clinical application requires further exploration to establish its safety, efficacy, and optimal use strategies (165, 166).

In conclusion, while topical eye drops remain the cornerstone of DED treatment, alternative drug delivery methods such as subconjunctival injection, implants, iontophoresis, and microneedle delivery offer new therapeutic options, particularly for refractory or severe DED cases. These methods can enhance drug bioavailability, provide more sustained therapeutic effects, and reduce the burden of frequent dosing. However, these novel delivery routes also come with potential side effects and technical challenges that require careful consideration in clinical practice. Future research should continue to explore the efficacy, safety, and optimal application strategies of these drug delivery methods in DED to provide more individualized and effective treatment options for patients.

## 6 Personalized immunotherapy

### 6.1 Application of biomarkers in Dry Eye Disease

Diagnostic and monitoring biomarkers: Biomarkers play a crucial role in diagnosing and monitoring DED. Recently, various biomarkers in the tear film have been found to closely correlate with the severity of DED. These biomarkers include cytokines such as IL-6, IL-8, TNF-α, and IL-17, which are significantly elevated in the tears of DED patients and correlate strongly with tear film stability and ocular surface damage indicators. Detecting these biomarkers allows for more accurate assessment of DED severity and monitoring of treatment efficacy (167). Fang (2019) showed that tear proteomics and bioinformatics analysis can effectively predict the effects of topical steroid treatment and dryness stress in DED patients. This randomized, double-blind, controlled clinical trial with 41 patients validated the application of tear protein biomarkers in DED diagnosis and treatment (168).

Biomarker-guided personalized treatment: Using biomarkers to guide personalized treatment is a significant advancement in DED management. By detecting biomarkers in the patient’s tear film, personalized treatment plans can be formulated based on different etiologies and pathological mechanisms. For instance, anti-inflammatory treatments can be employed for inflammation-mediated DED, while osmoprotectants can be used for tear film hyperosmolarity. Biomarkers can also monitor treatment efficacy and allow prompt adjustment of therapeutic strategies (169). Roy et al. (2023) emphasized the importance of validated biomarkers and objective indicators in clinical research and patient care for DED. They noted that biomarker development provides significant opportunities for implementing personalized medicine (170).

### 6.2 Precision medicine

Precision medicine leverages multi-omics data—including genomics, epigenomics, transcriptomics, proteomics, and metabolomics—to provide personalized treatment plans based on patient characteristics. This approach helps identify different disease subtypes and specific therapeutic targets in DED, thereby enhancing treatment efficacy and safety. For example, multi-omics data analysis reveals key pathways and molecular mechanisms associated with DED, leading to the development of targeted therapies (171). Inomata et al. (2020) proposed that by analyzing medical big data and mobile health applications, personalized treatment plans for chronic diseases like DED can be developed. This data provides a solid foundation for personalized and precision medicine treatments (169).

Several clinical studies have explored the application of precision medicine in DED. Artificial intelligence and machine learning techniques can extract valuable information from vast biomedical data sets, aiding in early disease diagnosis and personalized treatment. Analyzing patient multi-omics data can better predict treatment responses and outcomes, allowing for the creation of more precise treatment plans (172). Research indicates that soluble factors in tears, such as cytokines and chemokines, are excellent surrogate markers for disease severity. These markers can be used for disease classification and formulating treatment strategies. By non-invasively collecting and quantitatively measuring these soluble factors, tears become the optimal sample for molecular stratification of DED patients and monitoring treatment responses (152).

## 7 Future research directions and challenges

### 7.1 Research hotspots in novel immunotherapies

#### 7.1.1 Discovery and validation of new targets

In recent years, numerous new immunotherapy targets have emerged in DED research. For example, studies have shown that blocking reactive aldehyde species (RASP) can significantly alleviate DED symptoms and signs. The RASP inhibitor Reproxalap demonstrated rapid and broad symptom control and significantly improved fluorescein staining results in clinical trials, indicating that RASP inhibition could be an effective target for DED treatment (173). Additionally, the discovery of anti-citrullinated protein antibodies (ACPAs) in the tears of DED patients offers new insights for developing novel immunotherapies. ACPAs are associated with ocular surface disease, and human immunoglobulin eye drops can significantly reduce ACPA-induced ocular surface inflammation and disease (174).

#### 7.1.2 Multi-target combination therapy strategies

Multi-target combination therapies are considered potential avenues for improving DED treatment outcomes. Recent studies have shown that combining light therapies (intense pulsed light (IPL) and low-level light therapy (LLLT)) can significantly improve clinical indicators and molecular markers in patients with chronic meibomian gland dysfunction (MGD) and DED. These combined light therapies have demonstrated significant symptom relief and reduced inflammation by decreasing multiple inflammatory factors in tears, such as IL-1β, IL-17F, and MMP-9 (175). Furthermore, nanotechnology has been used to enhance the delivery efficiency of drugs like cyclosporine A, achieving the benefits of multi-target combination therapy through integration with other treatments (176).

### 7.2 Challenges in clinical translation

#### 7.2.1 Balancing safety and efficacy

Balancing safety and efficacy is a critical challenge in developing new immunotherapies. Although many new therapies show promising efficacy in early clinical trials, their long-term safety needs further validation. For instance, OTX-101, a novel cyclosporine A nanoemulsion, demonstrated significant efficacy and good tolerance in treating DED in clinical trials. However, its long-term safety requires further investigation (177). Similarly, early trials of Reproxalap showed rapid improvement in dry eye symptoms, but its long-term safety and efficacy need further assessment (173).

#### 7.2.2 Long-term follow-up and efficacy evaluation

Long-term follow-up and efficacy evaluation are crucial in the clinical translation of new immunotherapies. Many new therapies exhibit significant short-term efficacy, but their long-term effects and sustainability must be verified through extended follow-up studies. For example, CyclASol, a novel water-free cyclosporine formulation, demonstrated early efficacy in initial clinical trials, but its long-term follow-up results need further investigation (70). Additionally, research should focus on patients’ long-term adherence and the sustainability of treatment effects to comprehensively evaluate the clinical value of new therapies.

## 8 Summary

Recent advances in immunotherapy for DED have shown significant progress in several key areas. New biologics, such as Reproxalap, have demonstrated rapid symptom control and improved ocular surface inflammation. Stem cell therapies, particularly MSCs, offer promising anti-inflammatory, tissue repair, and immunomodulatory effects. MSC-derived exosomes provide a cell-free therapeutic option, further enhancing the potential for targeted immunomodulation. Nanotechnology has also improved drug delivery by increasing bioavailability and retention time. Future research should focus on multi-targeted combination therapies, precision medicine, and personalized treatment using multi-omics data to enhance efficacy and safety. Additionally, developing new drug delivery systems, long-term treatments, and continuous monitoring using real-time biomarkers will improve treatment outcomes and patient compliance. These advancements hold the potential to significantly improve the effectiveness and safety of DED treatments, ultimately enhancing patient quality of life.
